# Rare cytogenetic abnormalities and their clinical relevance in pediatric acute leukemia of Saudi Arabian population

**DOI:** 10.1186/s13039-019-0454-0

**Published:** 2019-10-11

**Authors:** Nawaf Alkhayat, Ghaleb Elyamany, Yasser Elborai, Qanita Sedick, Mohammad Alshahrani, Omar Al Sharif, Abdulmalik Alenezy, Amjad Hammdan, Hatem Elghezal, Omar Alsuhaibani, Mansour S. Aljabry, May AlMoshary, Eman Al Mussaed

**Affiliations:** 1 Department of Pediatric Hematology/Oncology, Prince Sultan Military Medical City, Riyadh, Saudi Arabia; 2 Department of Central Military Laboratory and Blood Bank, Prince Sultan Military Medical City, Riyadh, Saudi Arabia; 3 Department of Pediatric Oncology, National Cancer Institute, Cairo University, Cairo, Egypt; 4 Department of Pathology, Hematology unit, King Khalid University Hospital, King Saud University, Riyadh, Kingdom of Saudi Arabia; 5 Basic Science Department, College of Medicine, Princess Nourah Bint Abdulrahman University, Riyadh, Kingdom of Saudi Arabia

**Keywords:** Acute leukemia, Rare chromosomal abnormalities, Outcome, Pediatric

## Abstract

**Background:**

Childhood Acute Leukemia (AL) is characterized by recurrent genetic aberrations in 60% of AML cases and 90% of ALL cases. Insufficient data exists of rare cytogenetic abnormalities in AL. Therefore, we tested rare cytogenetic abnormalities occurring in childhood AL and its effect on clinical prognosis in patients diagnosed at our institution from 2010 to 2017.

**Results:**

Among 150 cases of AL, we detected 9 cases with rare chromosomal abnormalities. We found two hypodiploid (2n-) cases: 2n-,t (5;14)(q31;q32) and t (3;11;19)(q21;q23;q13.1) in ALL patients. AML patients showed t (7;14)(q22;q32), t (11;17)(p15;q21), t (11;20) (p15;q11), t (12;17)(q15;q23) and t (11;20)(p15;q11). Both t (1;15)(q10;q10) and t (17;19)(q21;p13.3) occurred in a case with biphenotypic AL. Complete remission (CR) status was attained in 3 patients and 6 patients never attained CR or relapsed/demised.

**Conclusion:**

The study highlighted that rare cytogenetic abnormalities are associated with a poor prognosis. This finding is not well reported in the literature suggesting that ongoing cytogenetic studies for rare abnormalities associated with pediatric leukaemia are warranted.

## Background

Leukemia is the most common form of pediatric cancer occurring in one-third of childhood malignancies [1].

Acute Leukemia (AL) is a clonal hematological disorder which occurs following a genetic alteration. Acute Lymphoblastic Leukemia (ALL) occurs more frequently in the pediatric age group compared to Acute Myeloid Leukemia (AML) [1].

Cytogenetic investigations using G-banding and fluorescence in-situ hybridization (FISH) is an essential tool for diagnosis, prognosis and targeted therapy. Chromosomal abnormalities are grouped into three prognostic categories: favorable, intermediate and adverse [2]. Some of these abnormalities is common and others are rare. Rare cytogenetic abnormalities that have been described in the literature include aberrations of chromosomes 3, del (5q),- 5 and − 7 [3].

While common recurrent cytogenetic abnormalities in AL have been well risk-stratified, the prognostic significance of many rare cytogenetic abnormalities in ALL and AML remains uncertain [4].

There is a paucity of data on the prevalence and clinical outcome of rare cytogenetic abnormalities in the Saudi Arabian population. In this study we have examined rare cytogenetic abnormalities in childhood AL and the clinical outcome.

## Materials and methods

### Patients

We reviewed 150 cases with a diagnosis of childhood pediatric acute leukemia at Prince Sultan Military Medical City in Riyadh, Saudi Arabia from 2010 to 2017. The diagnosis in all cases was based on morphology, flow cytometry, immunohistochemistry and genetic studies. This study included patients between 1 and 18 years of age (pediatric age group in Saudi Arabia). The medical records were reviewed for all data (Tables 1 and 2).

**Table 1:** Demographic data cases 1–5

Parameter	Case 1	Case 2	Case 3	Case 4	Case 5
Age (years)	4	7	11	18	3
Gender	Female	Female	Male	Male	Male
Clinical	Cervical Lymphadenopathy and leucocytosis	Fever, abdominal distention, hepatosplenomegaly	Vomiting, diarrhoea, lower limb weakness	Epistaxis, ecchymosis skin lesions	Fever, lymphadenopathy and hepatomegaly
WBC (10^9^/L)	137	18	100,8	221	18.8
HB (g/dl)	5.2	4.8	7.2	11	8.7
PLT (X109/L)	9	53	109	34	239
PB blasts	80%	60%	No blasts	40%	6%
Bone marrow aspirate	Hypercellular Blasts 90% B-ALL phenotype	Hypercellular Blasts 80% B-ALL phenotype	40% blasts and 50% eosinophils	40% blasts on PB and 70% blasts on BM	Hypercellular Blasts 75% Myelomonocytic proliferation with M4 AML
Blasts	90%	60%	40%	70%	75%
Disease	B-ALL	B-ALL	B-ALL	T-ALL	AML FAB M4
Cytogenetic analysis (karyotype/FISH)	46, XX,1-, 8-, 9-, 11-, 12-, 19-, and 22- in 80% of cells)	2n-,44, XX,- 4, −8). t (12; 17). *ETV/RUNX 1.* Loss of der (12-) MYC gene rearrangement.	t (5;14) Negative: *PDGFRA PDGFRB FGFR1*	46,XY,t(3;11;19) (q21; q23; q13.1). *MLL g*ene rearranged; extra *MYC* gene	48,XY,+ 8,+ 8,t(11;17) (p15;q21) [14]/47, idem,-Y
Chemotherapy	Very high risk ALL chemotherapy protocol (COG AALL0031) and intensive consolidation	Standard risk chemotherapy protocol (COG AALL0331).	high-risk protocol COG AALL0232 with high dose methotrexate for maintenance	Dana Farber then FLAG-Ida salvage chemotherapy high dose	MRC AML12 protocol.
Survival	Partial remission	Complete remission status achieved	Complete remission	Relapsed and demised	Death due to multiorgan failure

**Table 2:** Demographic data cases 6–9

Parameter	Case 6	Case 7	Case 8	Case 9
Age (years)	14	18	5	4
Gender	Male	Male	Male	Male
Clinical	Melena stools, fatigue	Leucocytosis, anaemia, thrombocytopenia no organomegaly	Generalized ecchymosis, bruises, epistaxis and hepatomegaly	Fever, malaise failure to thrive
WBC (109/L)	16.5	33	16.1	100
HB (g/dl)	10.1	8.2	6.7	7.8
PLT (X109/L)	20	67	14	101
PB blasts	30%	70%	40%	70%
Bone marrow aspirate	90% blasts with AML MO morphology	70% blasts	40% blasts With dysplasia	90% blasts comprised of two distinct populations
Blasts	90%	70%	40%	90%
Disease	AML MO	AML M2	AML M7	B/MYELOID
Cytogenetic analysis (karyotype/FISH)	46,XY,t(7;14) (q22;q32)	46,XY,t(11;20) (p15;q11), add(21)(p11)	t(12;17)(q15;q23), del(7)(p15), inv. (8)(q22q24) with(2+) and(19+)	46,XY,der(15)t(1;15) (q10;q10),der(17) t(17;19)(q21;p13.3)
Chemotherapy	Received induction (3 + 7) for AML then high risk MAC/G protocol	AML induction chemotherapy (3 + 7) protocol. Allogeneic stem cell transplant with steroid refractory graft vs host disease treated with ATG	MRC AML12 Protocol	Received 7 chemotherapy cycles
Survival	Refractory disease and demised secondary to chemotherapy side effects	Complete remission	Patient demised	Not attain remission Status. Relapsed for MUD transplant

### Cytogenetic analysis and FISH

Standard cytogenetic preparations were made from bone marrow and/or peripheral blood. Cytogenetic analysis was carried out on G-banding chromosomal preparations in a total of 20 metaphases. Karyotypes were interpreted and reported according to the International System for Cytogenetic Nomenclature [5].

Fluorescence in situ hybridization (FISH) was performed on double stranded DNA in fixed chromosomes using fluorescent probes which bind complementary sequences of mRNA in a sequence of hybridization steps to achieve signal amplification of the target which is viewed using a fluorescent microscope.

The panel of probes used to detect ALL specific abnormalities in our institute are *BCR*-*ABL*: t (9;22), *RUNX1*-*ETV6*: t (12;21), *MLL* gene rearrangements:(11q23), *MYC* gene rearrangements:(8q24) and *TCF3*/*PBX1*: t (1;19).

The multiprobe AML panel includes: *RUNX1*/*RUNX1T1*: t (8;21), *PML* /*RARα*: t (15:17), *CBFβ* gene break apart: 16q22, *MLL* gene break apart, *TP53* gene and the cen(8, 22q11.2).

## Results

**Fig. 1:**
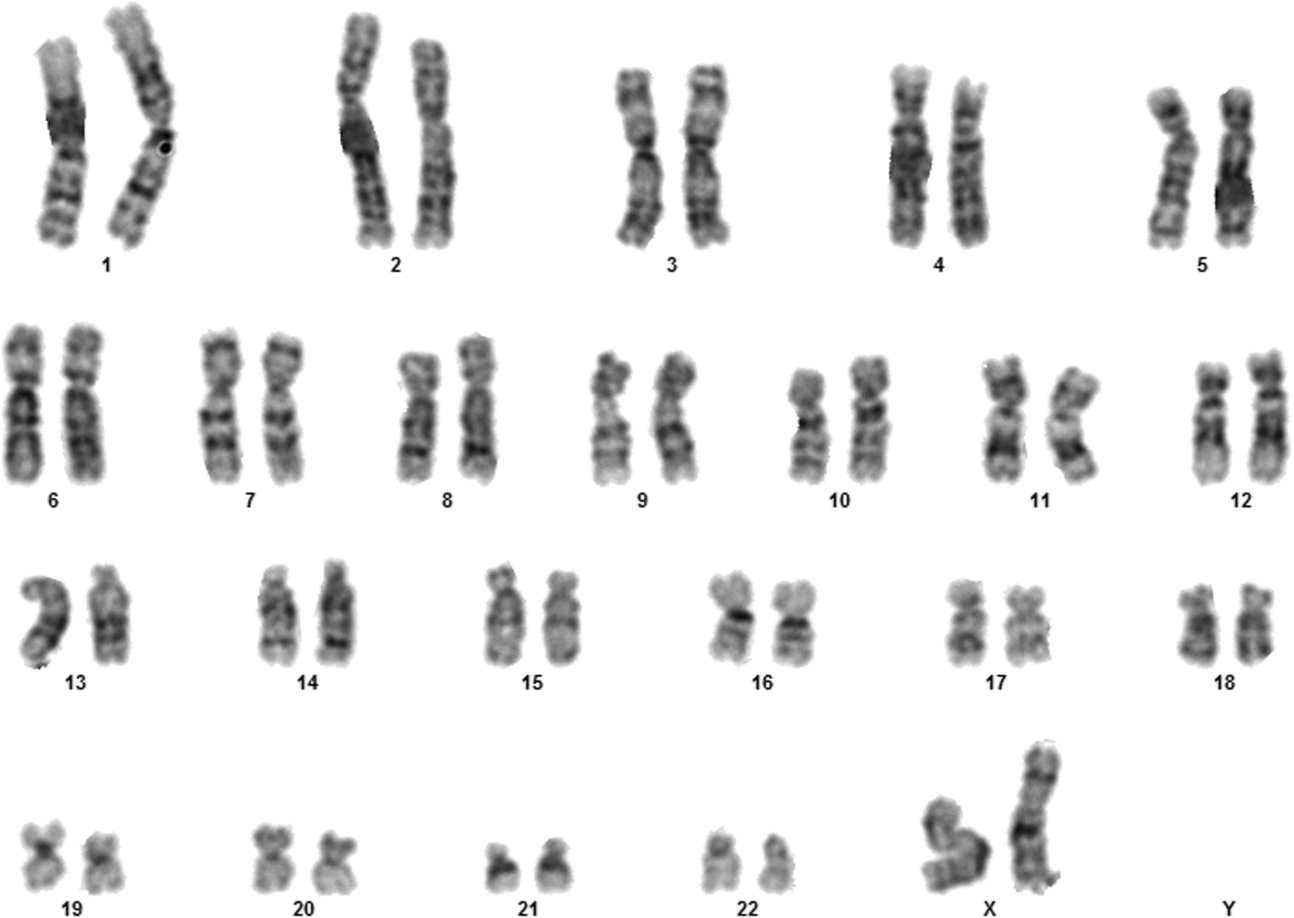
Case 1 showing normal karyotype

**Fig. 2:**
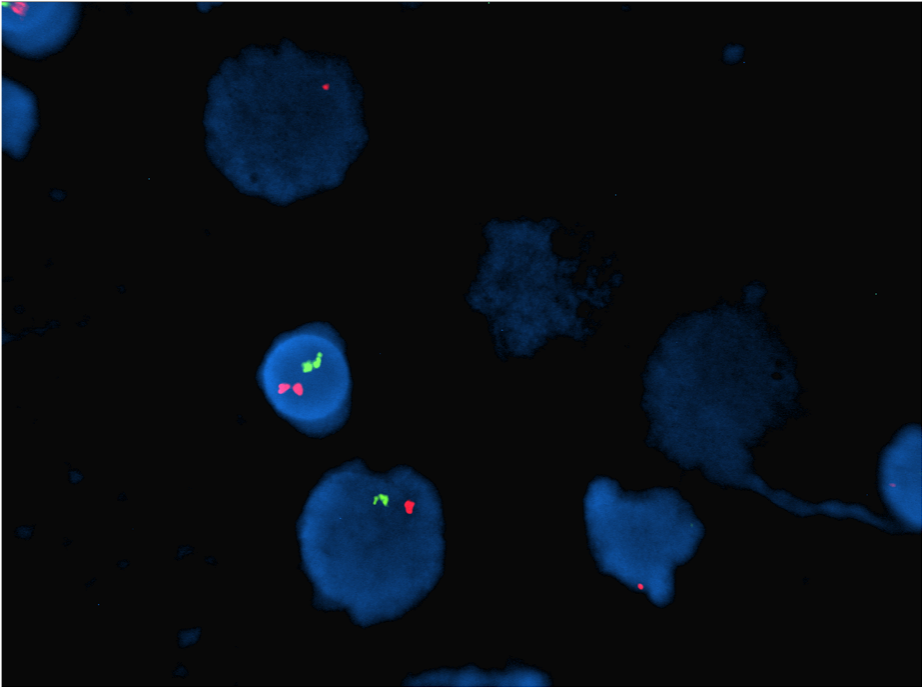
Case 1 FISH showing 2n- in *BCR-ABL* probe (monosomy 9 and 22)

**Fig. 3:**
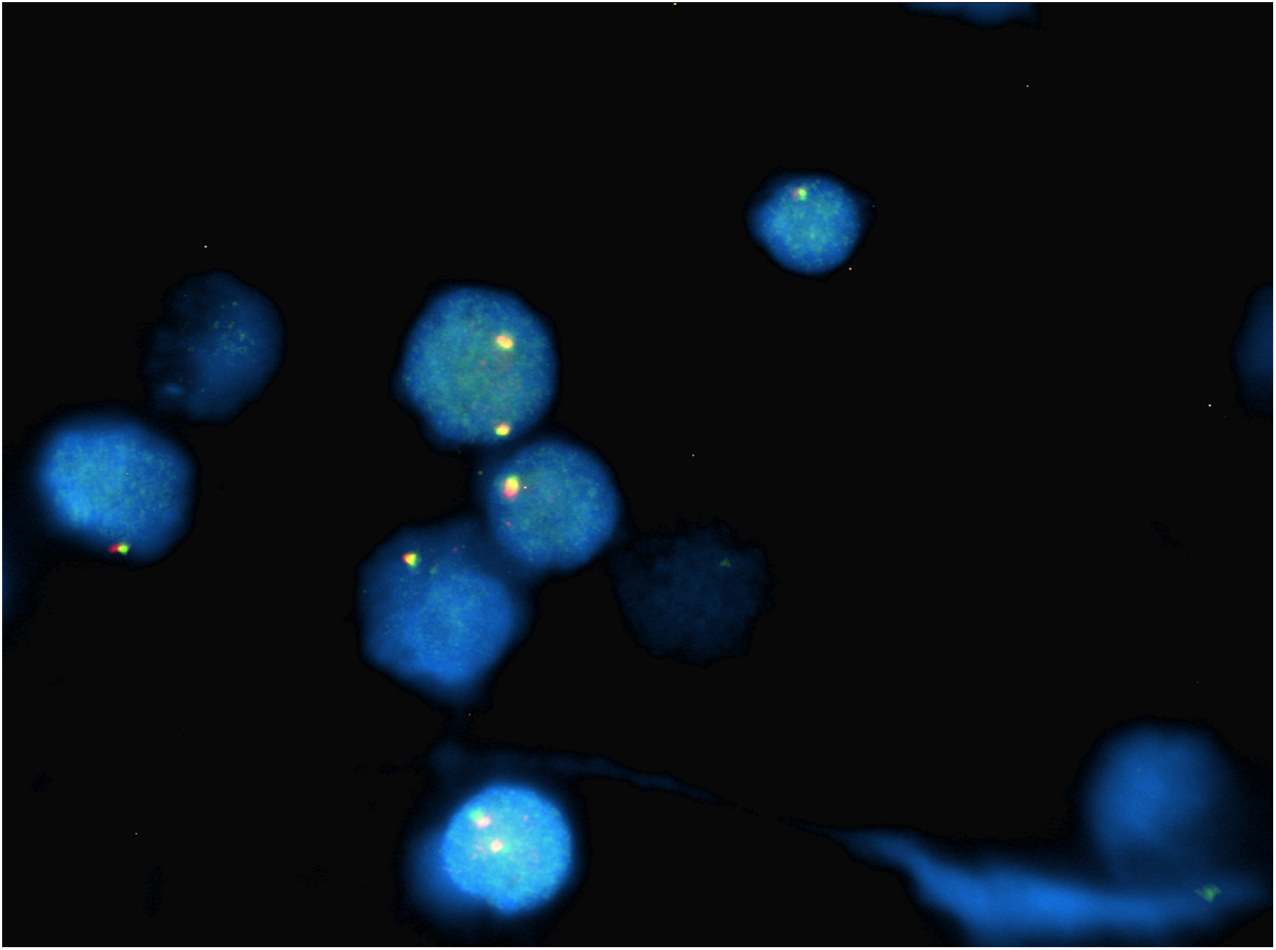
Case 1 FISH showing 2n- in *MYC* probe (monosomy 8)

**Fig. 4:**
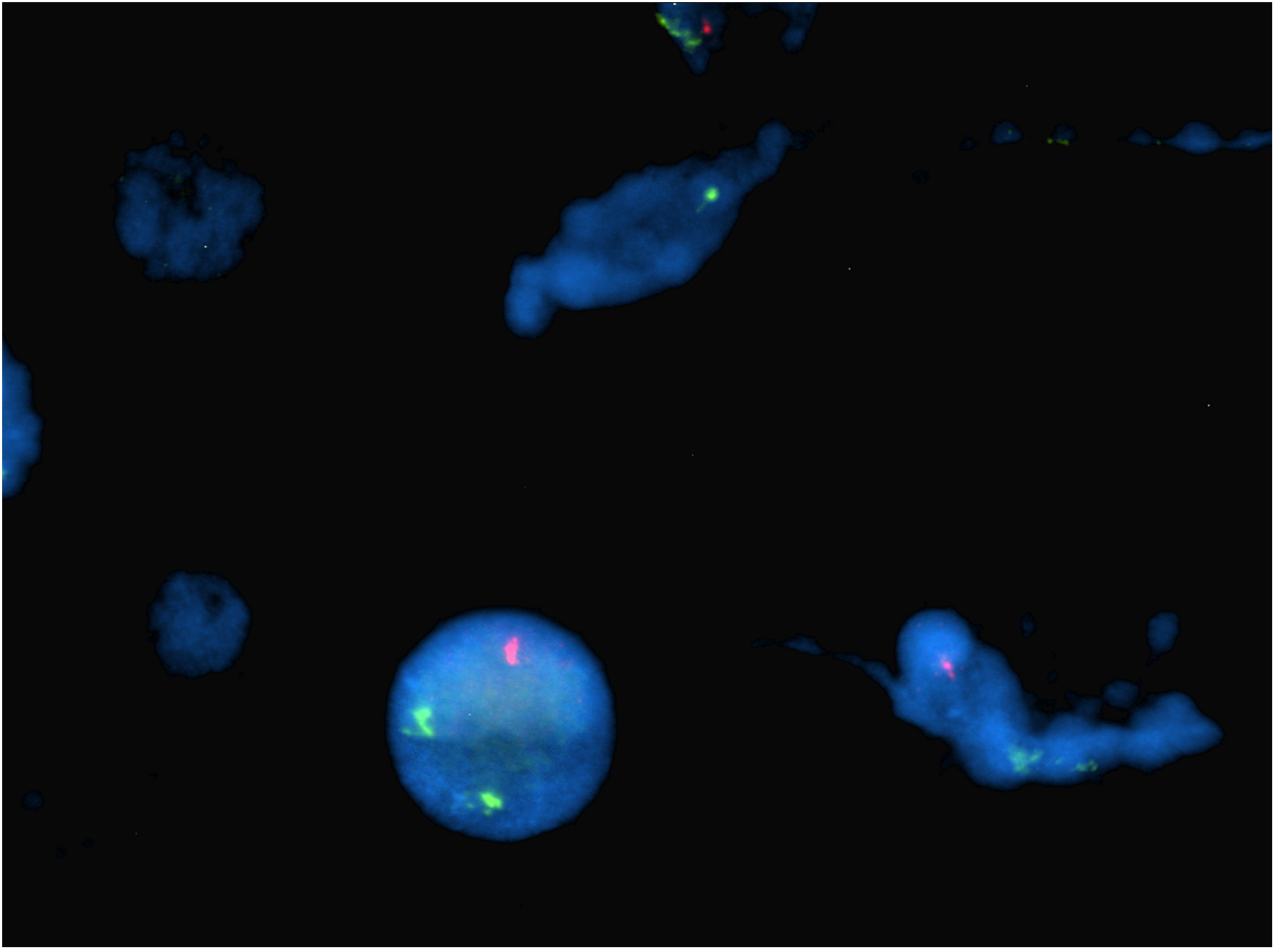
Case 1 FISH showing 2n- in *MLL* probe

**Fig. 5:**
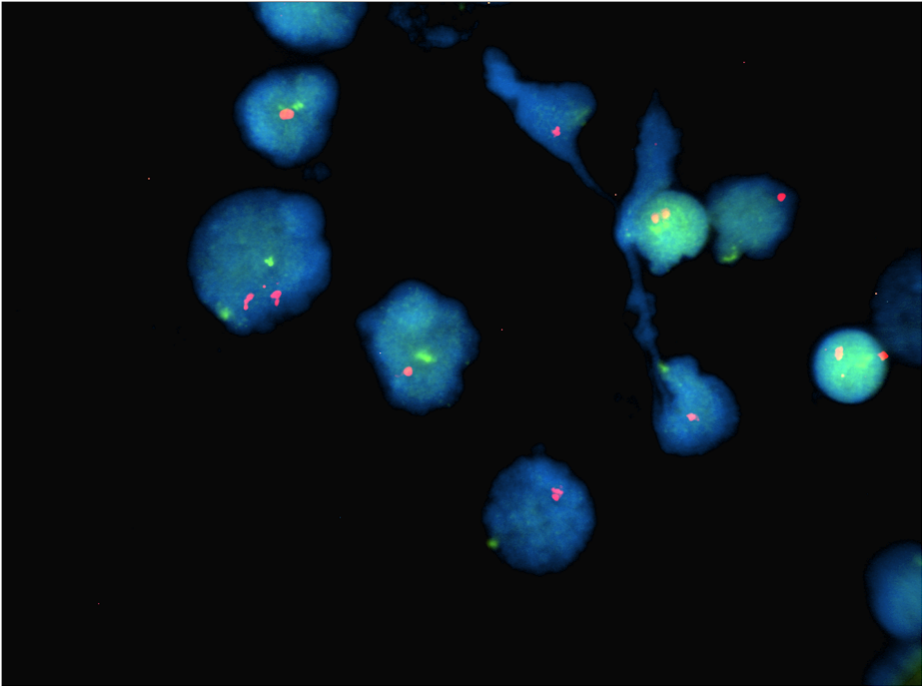
Case 1 FISH showing 2n- in *ETV6-RUNX1* probe

**Fig. 6:**
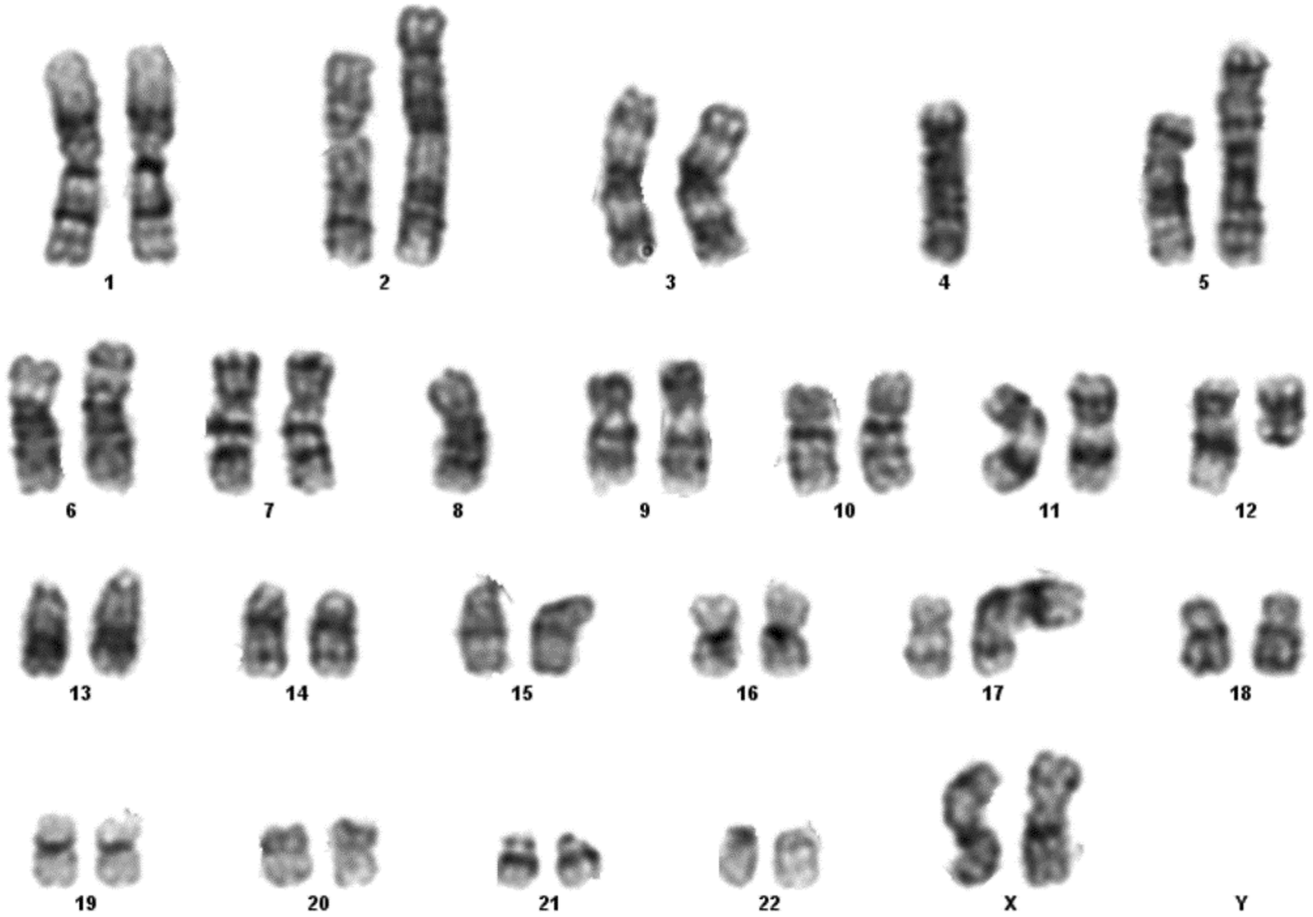
Case 2 showing complex karyotype: 46,XX,-4,-8,t (12;17)

**Fig. 7:**
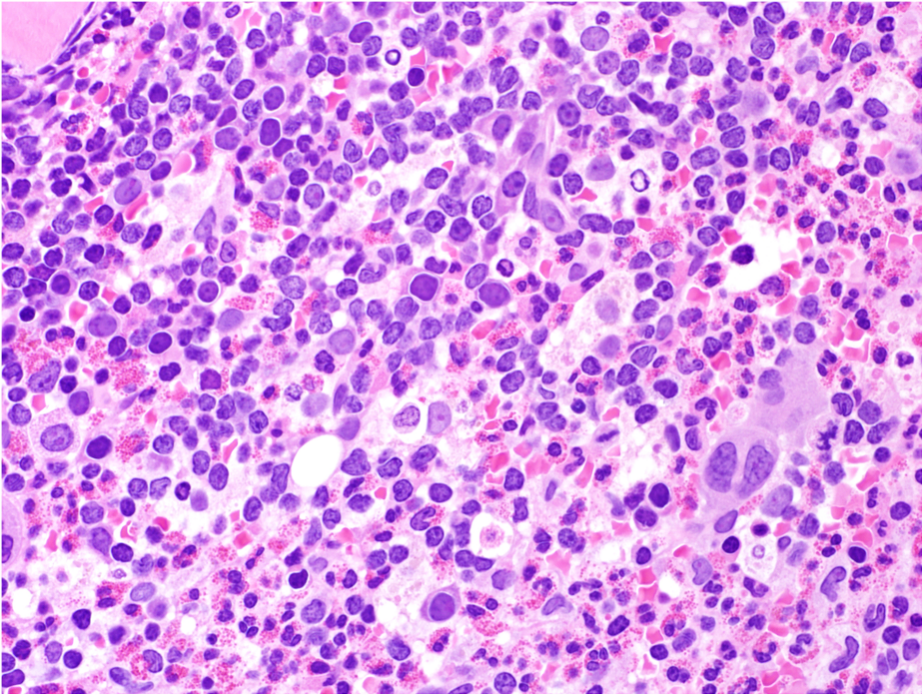
Case 3 hematoxylin and eosin stain shows prominent eosinophilia

In our cohort of 150 cases of acute leukemia, we detected 9 cases with rare non-recurrent chromosomal abnormalities of which 4 cases were ALL, 4 cases were AML and one case was biphenotypic AL (B/Myeloid). Two cases with hypodiploidy (2n-), t (5;14) (q31;q32) and t (3;11;19)(q21;q23;q13.1) were detected in ALL. The AML patients were found to harbor t (7;14) (q22;q32), t (11;17)(p15;q21), t (11;20)(p15;q11), t (12;17)(q15;q23) and t (11;20)(p15;q11). Both t (1; 15) (q10; q10) and t (17; 19) (q21; p13.3) were detected in the case with biphenotypic AL. The demographic, hematological and cytogenetic data of these 9 cases are summarized in Table 1& Table 2 and Figs. 1, 2, 3, 4, 5, 6, 7. Complete remission (CR) status was achieved in 3 patients. The remaining 6 patients never attained CR, relapsed or demised.

### Acute lymphoblastic leukemia cases

#### Case 1

This 4 year old B-ALL patient was negative for ALL panel specific abnormalities with a normal female karyotype (46, XX) (Fig. 1). Hypodiploidy (2n-) with loss of -1, -8, -9, -11,-12,-19 and -22 was detected in 80% of the studied cells by FISH (Figs. 2, 3, 4, 5). Thereafter, cryptic abnormalities were identified (not detected by the initial karyotyping (Fig. 1). The interesting finding in this case was that the diagnostic karyotype was normal but the FISH showed 2n-. This indicated that FISH revealed the cryptic cytogenetic abnormality which was not detected by GTG-banding karyotype.

The patient was classified on very high risk ALL chemotherapy protocol (COG AALL0031). During induction chemotherapy the patient developed a gluteal ulcer and recurrent infections with positive blood cultures which we treated with antibiotic therapy. The post induction BM aspirate revealed 6% of blasts with the immunophenotype presentation compatible with partial remission. The patient received 2 weeks of extended induction chemotherapy. The BM aspirate on day 43 showed morphological remission. Cytogenetics was negative for all detected tumoral clones except for the 2n- which persisted. The patient then received intensified consolidation phase chemotherapy and is currently awaiting BM transplant.

#### Case 2

This 7 year old B-ALL patient harbored the classical *ETV6* /*RUNX1* rearrangement in the majority of analyzed cells. However, a clonal evolution with loss of der (12)- and the *MYC* gene rearrangement was detected in 20% of cells. The karyotype showed 44, XX; del (4); del (8) and t (12; 17) (p13; q21) (Fig. 6).

The patient was classified on standard risk chemotherapy protocol (COG AALL0331). The post induction BMA showed CR. The clinical decision was to continue the chemotherapy protocol in consideration of the mild 2n- . Hypodiploidy (2n) - < 45 chromosomes is uncommon. Despite improved treatment outcome of childhood ALL, patients with hypodiploid ALL have a dismal prognosis [6–8].

#### Case 3

**Fig. 8:**
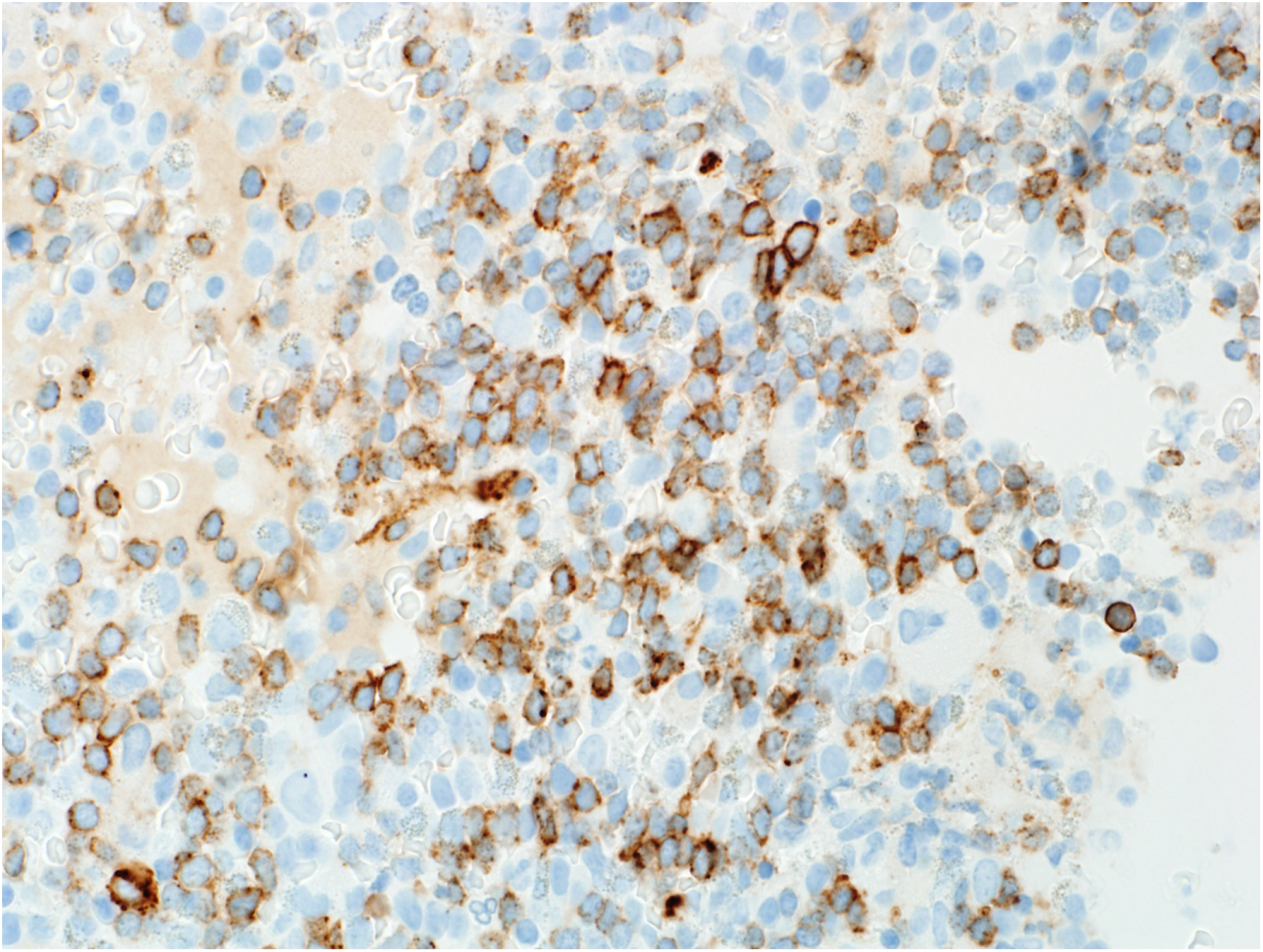
Case 3 shows CD34+ blasts on immunohistochemistry

This 11 year old patient presented with vomiting, diarrhea and generalized weakness for 3 weeks. The full blood count detected leukocytosis with marked eosinophilia. The BM was hypercellular with eosinophilia (50%) (Fig. 7) and blasts (40%) (Fig. 8) with B-ALL immunophenotype. The cytogenetic and molecular analysis detected t (5; 14) (q31; q32) by FISH. RT-PCR was negative for *PDGFRA*, *PDGFRB*, and *FGFR1* gene abnormalities. We diagnosed the patient with concurrent B-ALL and hypereosinophilia.

The patient was classified on steroid therapy and on high-risk chemotherapy at the time of diagnosis. Post induction chemotherapy analysis showed morphological(< 3% blasts/ no eosinophil’s in the BM) and molecular (negative IGH gene rearrangements) remission. The patient is currently in CR status on high dose methotrexate therapy for maintenance.

The t (5, 14) in association with eosinophilia has not been frequently reported in the literature. A single case report of a 6 year old boy presenting with hypereosinophilia and associated Loeffler endocarditis has been previously recorded [8]. Three months following his initial hypereosinophilia this patient developed cutaneous B-lymphoblastic lymphoma. Re-analysis of apparently uninvolved BM revealed a single, previously unidentified.

t (5; 14) (q31; q32) positive cell. *IL3* / *IGH* @ fusion were demonstrated in cutaneous lymphoma cells. Our patient also showed the *IL3/IGH* gene translocation strengthening the association of *IL3* hypersecretion and hypereosinophilia [8].

#### Case 4

**Fig. 9:**
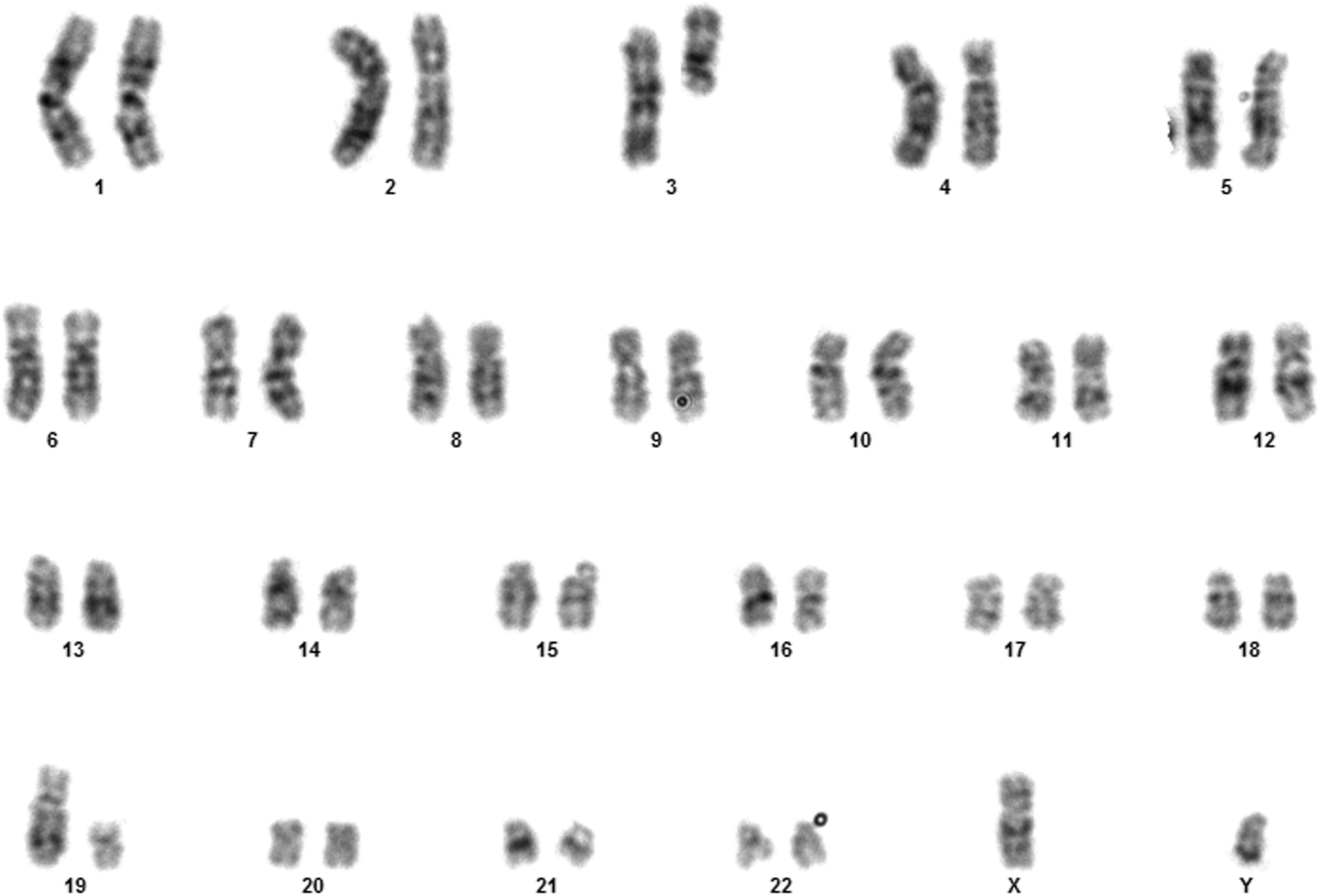
Case 4 Karyotype showing 46, XY, t (3;11;19)

**Fig. 10:**
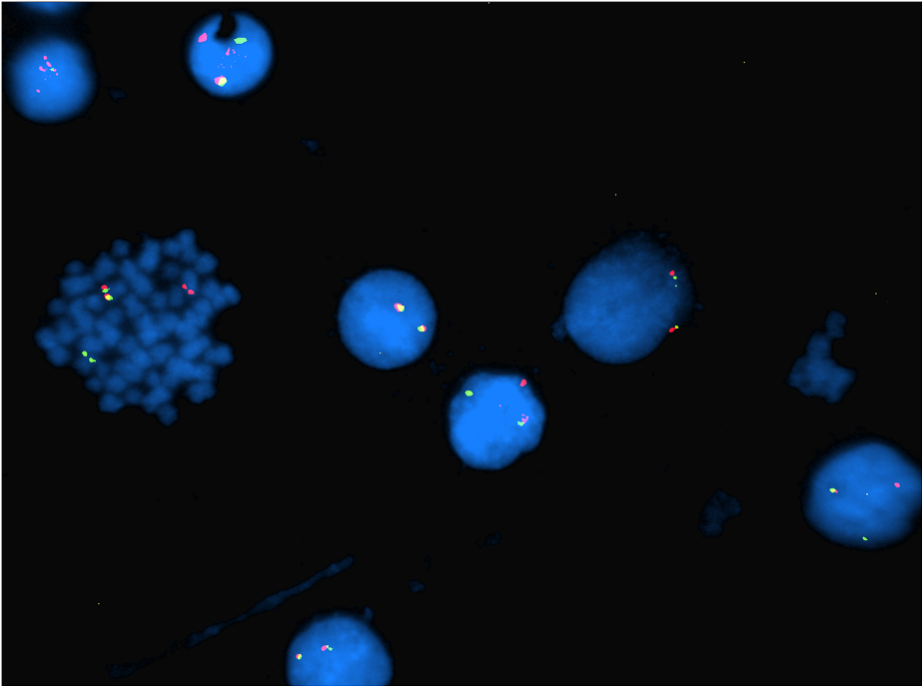
Case 4 FISH showing *MLL* gene rearrangement

**Fig. 11:**
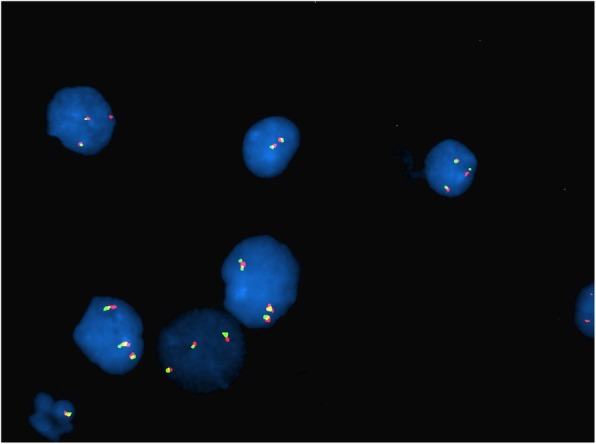
Case 4 FISH showing extra copy of *MYC* gene

This 18 year old T-ALL patient presented with the typical T cell immunophenotype on 40% of blasts (CD45 dim, CD4, CD8, CD7, CD5, CD2, CD38, CD34, cCD3). The karyotype was 46,XY; t (3;11;19)(q21;q23;q13.1) (Fig. 9). FISH was positive for *MLL* gene rearrangement (Fig. 10). An extra copy of the *MYC* gene was detected in 40% of the studied cells (Fig. 11).

The patient was classified on DANA FARBER protocol but did not respond. FLAG-IDA salvage chemotherapy high dose was started. The patient, however, never attained CR and subsequently demised. To the best of our knowledge, this is the first case reported in the literature harboring this complex translocation.

### Acute myeloid leukemia cases

#### Case 5

**Fig. 12:**
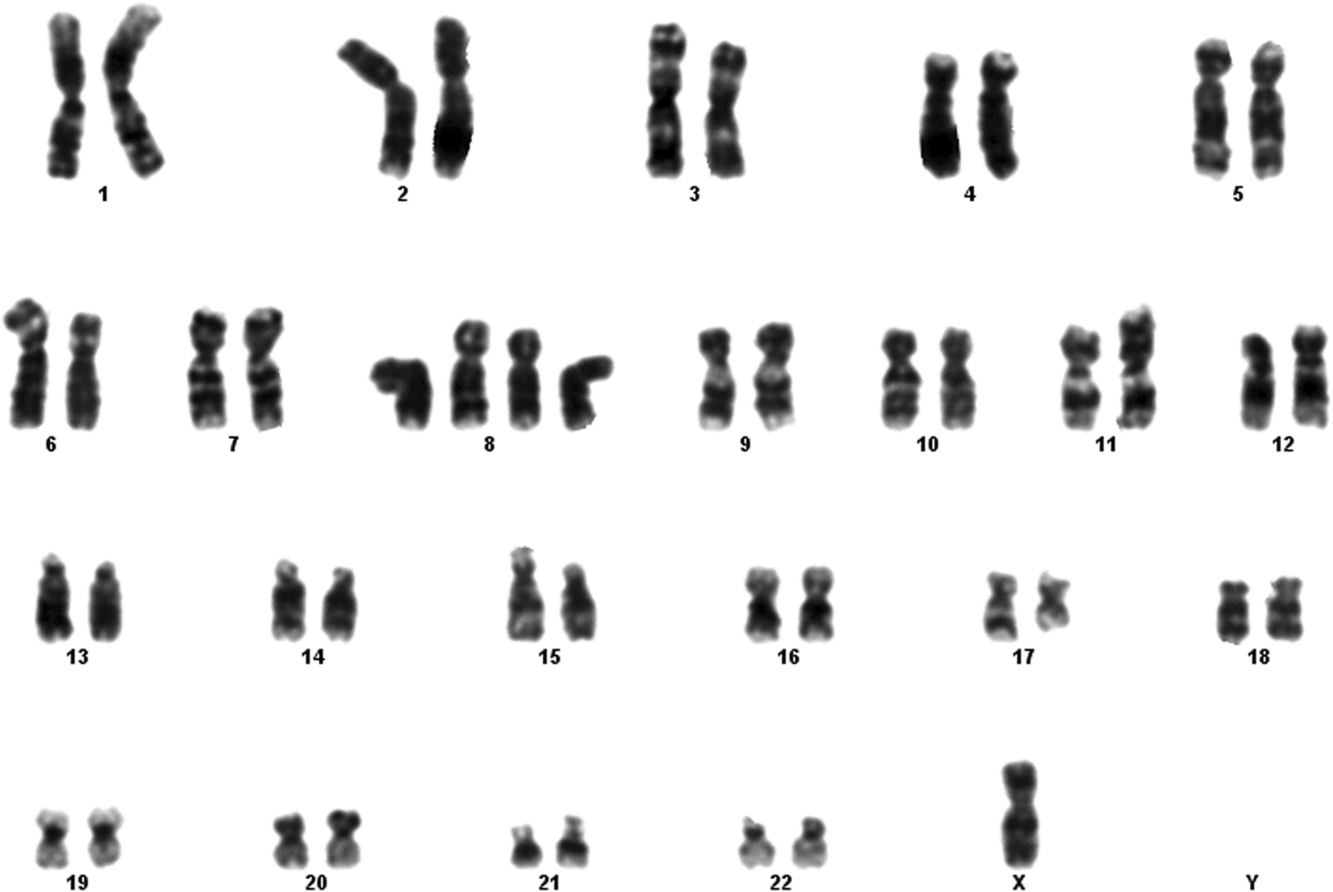
Case 5 Karyotype shows t (11;17), tetrasomy of chromosome 8 and extra copy of *MYC* gene

This 3 year old AML M4 patient showed t (11; 17) (p15; q21), tetrasomy (4n) of chromosome 8 and two extra copies of *MYC* in 85 and 70% of the studied cells (Fig. 12).

The patient was classified on the first cycle of MRC AML12 protocol. On day 5 post chemotherapy the patient developed neutropenia and persistent high grade fever. The patient was given Vancomycin and Amikacin following blood cultures and Meropenem for a urinary tract infection. Prophylactic fluconazole was started. On the final chemotherapy cycle the patient developed bloody diarrhea and abdominal distention. The abdominal ultrasound and CT Abdomen revealed a severe typhilitis. Despite intensive care support, the patient demised following cardiopulmonary arrest and multi-organ failure one month after admission.

Only 3 cases of pediatric AML with the t (11; 17) (p15; q21) have been previously reported: two AML M4 cases (aged 3 and 4 years) one AML M0 case [9–11]. Another MDS case with isolated t (11; 17) (p15; q21) after neuroblastoma chemotherapy has been reported in an 8 years old girl [12]. In adults, the translocation has been reported in one case [12].

#### Case 6

This 14 year old patient was diagnosed with Hodgkin’s Lymphoma (HL) stage 3-A and was in remission for 5 years. The patient was treated with ABVD and CHIVPP. He arrived at the Emergency Unit with the clinical symptoms of melena stools, fever, fatigue, lymphadenopathy and hepatosplenomegaly. The BM and immunophenotype was compatible with AML MO. A lymph node biopsy showed a myeloid sarcoma.

Chromosomal analysis detected the karyotype 46, XY, t (7; 14) (q22; q32). FISH was negative for AML panel specific abnormalities. After initiation of induction chemotherapy the patient developed persistent neutropenia with klebsiella infection and did not attain remission status. He was classified on high risk MAC/G protocol. He continued to have chemotherapy related side effects such as afebrile neutropenia, severe mucositis and multiple resistant bacterial and fungal infections. The patient failed to recover or attain remission status and subsequently demised. This is rare presentation of AML MO with t (7, 14) in a patient with previous HL.

Secondary leukemia’s as in this patient commonly manifest with abnormalities of chromosome 7 and 5, however, the t (7; 14) (q22; q32) commonly occurs in T-ALL and rarely in AML [13, 14].

#### Case 7

**Fig. 13:**
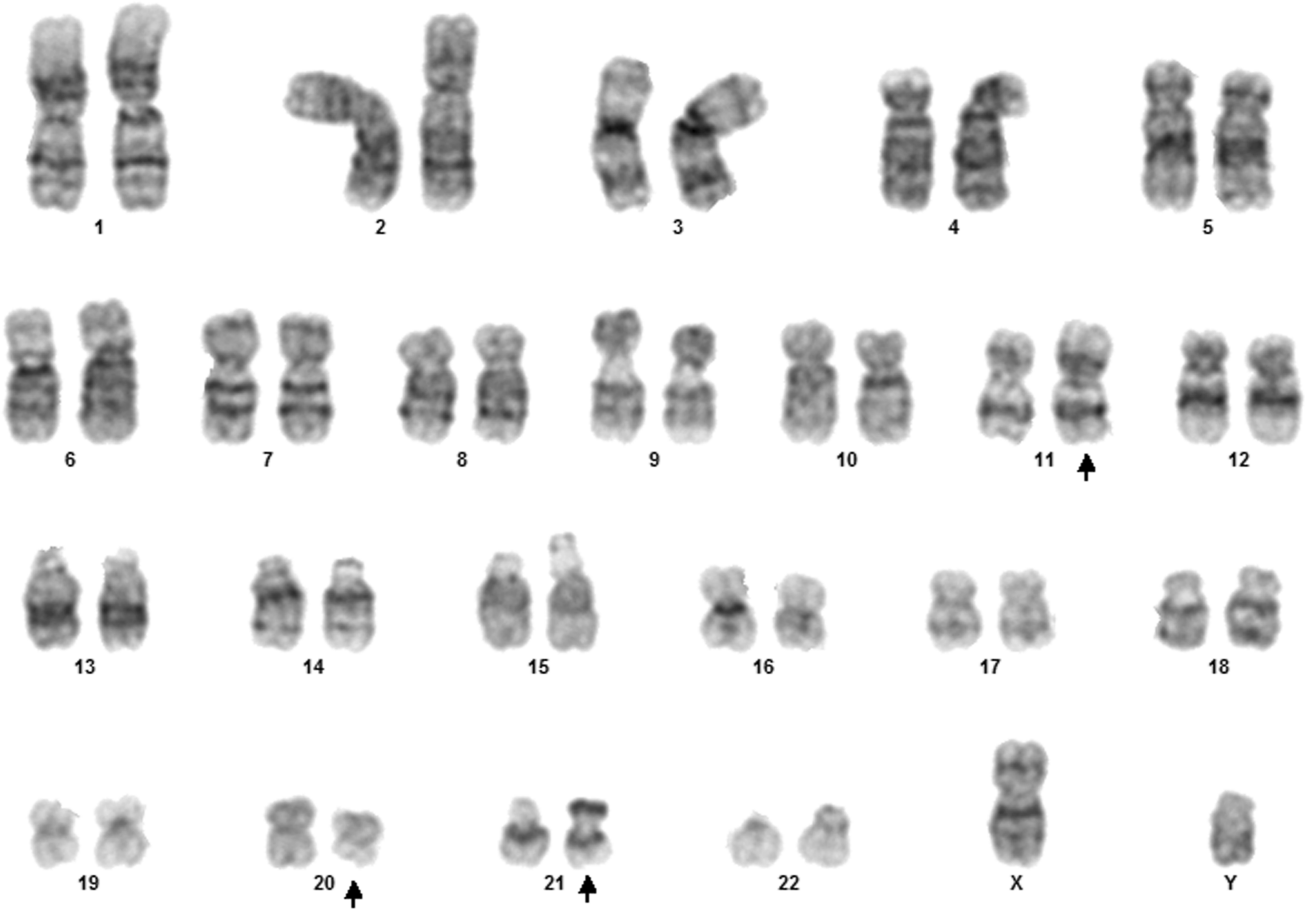
Case 7 Karyotype showing 46, XY, t (11;20),(21+)

This 18 year old patient was diagnosed as AML (M2) both morphologically and immunophenotypically. Aberrant expression of CD7 occurred on a cellular subpopulation. Cytogenetic analysis showed 46, XY; t (11, 20) (p15; q11) and add (21) (p11) (Fig. 13). The patient started the first cycle of AML induction chemotherapy (3 + 7) protocol and achieved CR.

This t (11; 20) (p15; q11) is a rare chromosomal translocation which has a poor prognosis [15, 16]. Our case responded well to 3 + 7 protocol (3 doses of Daunorubicin+ 7 days of cytosine arabinoside) and attained CR. The patient then had allogeneic stem cell transplant and later developed steroid refractory graft versus host disease which was treated with ATGA.

#### Case 8

**Fig. 14:**
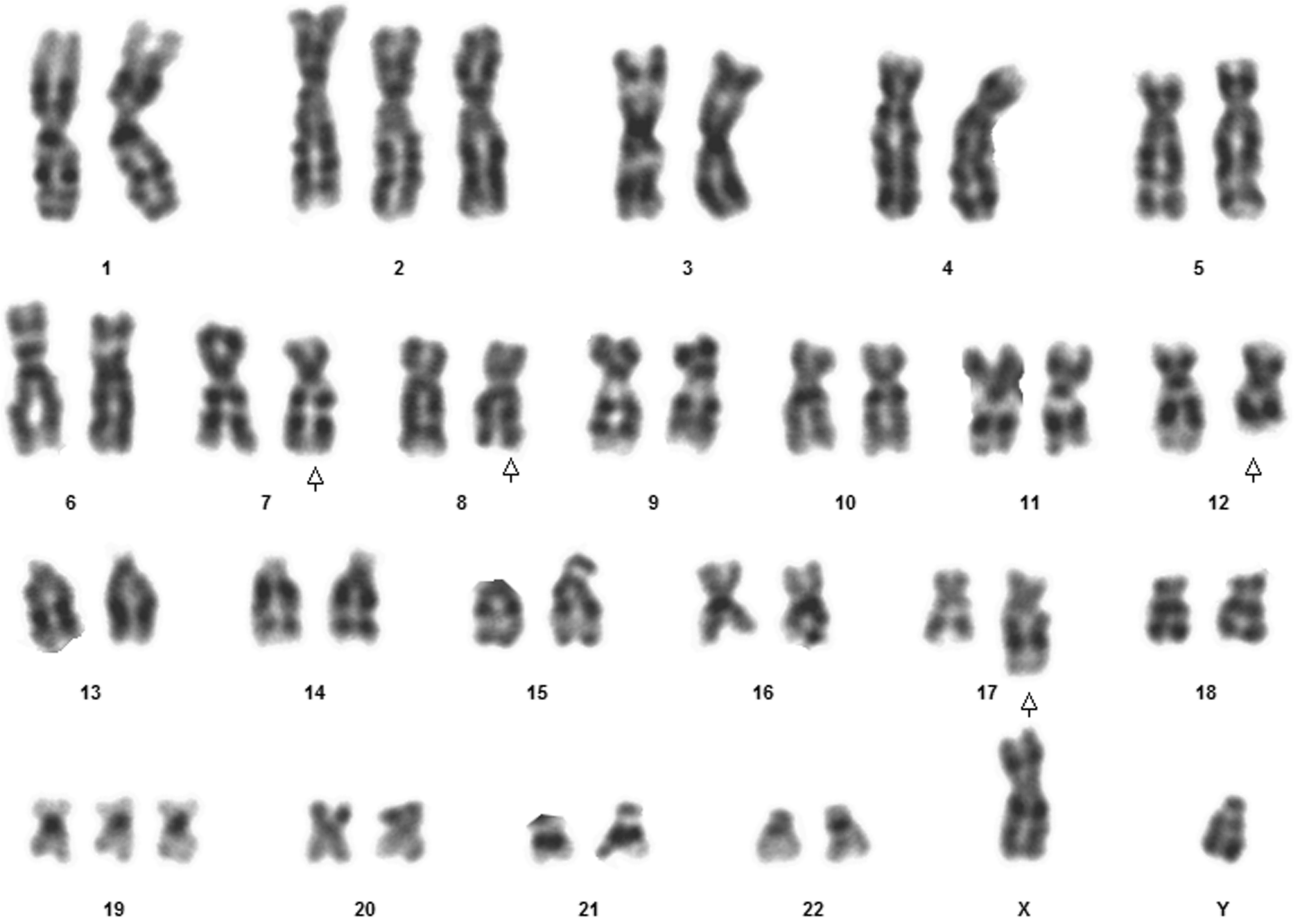
Case 8 showing complex Karyotype including t (12;17)

This 5 year old patient presented with anemia and thrombocytopenia. He received IVIG infusion as ITP (immune thrombocytopenic purpura) was suspected, but no improvement occurred. A BM aspirate immunophenotype was compatible with AML (FAB; M7). The cytogenetic analysis revealed a complex karyotype t (12;17)(q15;q23) and 48,XY,+ 2,del (7)(p15), inv. (8)(q22q24), t (12;17) (q15;q23) and trisomy 19. FISH reported *PML*/*RARA*; *RUNX*1 / *RUNX1T1*; (*5’CBFB*, *(3’CBFB,5’CBFB con 3’CBFB*) / *(5’MLL* (*3’MLL,5’MLL con 3’MLL)*. In addition, a tumoral clone with extra chromosome (2+) and (19+), del (7p), inv.(8) and t (12; 17) (Fig. 14) was detected.

The patient was treated on MRC AML12 Protocol but did not attain remission and subsequently demised. This very rare t (12; 17) has been reported in three adults and one child with secondary AML [17, 18]. Interestingly, the four published cases have been female and have additional aberrations. Our patient is male and the translocation is also part of a complex karyotype.

#### Case 9

This 4 year old patient was diagnosed as biphenotypic acute leukemia (B /Myeloid). Morphology of two morphologically diverse populations of cells immunophenotypically expressed myeloid markers (CD13, CD33 and MPO) and B cell markers (CD10, CD19, CD79a, and TdT).

The cytogenetic analysis revealed the presence of a cell line with der t (1;15)) (1q10; 15q10) and t (17q21; 19p13.3). The FISH panel was negative for all gene abnormalities.

We diagnosed a biphenotypic (B /Myeloid) leukemia with the rare t (1; 15) present in the AML clone and t (17; 19) present in the B-ALL clone. The patient was classified on MRC AML12 protocol. The post induction BM showed persistent disease (60% blasts). A second ADE was given and the BM showed a regenerating marrow with 5% clonal blasts. A third cycle of the protocol MACE and fourth cycle CLASP were given and samples were taken for matched unrelated donor transplant. During the fourth chemotherapy cycle, the patient developed septic shock and the protocol was changed to a fifth chemotherapy cycle MidAC. A month after completing this cycle, the patient presented with fever, bone aches and neutropenia with circulating blasts. The BM aspirate showed relapse with 60% blasts. The patient was classified on FLAG-IDA (the sixth chemotherapy protocol). However, the patient remained refractory. In addition, the patient developed febrile neutropenia and was started on antibiotics, antifungal therapy and a 7th course of chemotherapy. A matched unrelated donor transplant was planned by the treating physicians in view of the persistent refractory disease.

## Discussion

Cytogenetic investigations for chromosomal abnormalities are important tools for classification and prognostic determination in AL [19]. Response to chemotherapy in AL depends on the cytogenetic characteristics and patient’s age [2]. Leukemia’s with adverse cytogenetic abnormalities and older patients are associated with a poor prognosis [3].

While studies showing the prognostic significance of rare cytogenetic abnormalities in adults have been reported in large cohorts [5], there is a paucity of data showing this association in the pediatric population.

We studied a large series of pediatric patients with acute leukaemia in Saudi Arabia through GTG-banding and FISH techniques. We found that 9 of these cases harbored rare (non-recurrent) chromosomal abnormalities. We analyzed them and found correlations with regard to clinical presentation, outcome and cytogenetic abnormalities.

## Conclusion

Our results confirm that rare cytogenetic chromosomal abnormalities in pediatric AL are associated with a poor outcome. Data confirming these findings are sparsely reported in the literature suggesting that ongoing cytogenetic studies are warranted in larger groups of AL to identify rare and novel chromosomal abnormalities that may contribute to diagnosis and prognosis in pediatric patients with AL and help in the development of targeted therapeutic drugs.

## Data Availability

All data generated or analyzed during this study are included in this article and its supplementary material.

## Abbreviations

AL: Acute leukemia
ALL: Acute lymphocytic leukemia
AML: Acute myeloid leukemia
BM: Bone marrow
ChlVPP/ABVVP: Chlorambucil, vinblastine, procarbazine, doxorubicin, bleomycin, vincristine and etoposide
CLASP: Cytarabine plus L-asparaginase
COG: Children’s Oncology Group
CR: Complete remission
FISH: Fluorescence in situ hybridization
FLAG: Fludarabine + high dose AraC + GCSF
IDA: Idarubicin
MACE: Amsacrine + AraC +etoposide
MidAC: Mitoxantrone and AraC
MRC: Medical Research Council
RT-PCR: Reverse transcription polymerase chain reaction
